# Facial and semantic emotional interference: A pilot study on the behavioral and cortical responses to the dual valence association task

**DOI:** 10.1186/1744-9081-7-8

**Published:** 2011-04-13

**Authors:** Agustín Ibáñez, Esteban Hurtado, Rodrigo Riveros, Hugo Urquina, Juan F Cardona, Agustín Petroni, Alejandro Lobos-Infante, Joaquin Barutta, Sandra Baez, Facundo Manes

**Affiliations:** 1 Institute of Cognitive Neurology (INECO) and Institute of Neuroscience, Favaloro University, Argentina; 2 National Scientific and Technical Research Council (CONICET), Buenos Aires, Argentina; 3 Laboratory of Cognitive Neuroscience, Universidad Diego Portales, Santiago, Chile; 4 Pontificia Universidad Católica de Chile; 5 Laboratory of Epistemology and History of Medicine (LEPHIM), Instituto Universitario del Hospital Italiano de Buenos Aires, Argentina; 6 Integrative Neuroscience Laboratory, Physics Department, University of Buenos Aires, Argentina

**Keywords:** ERP, N170, LPP, IAT, DVAT, interference effects, valence, word, face

## Abstract

**Background:**

Integration of compatible or incompatible emotional valence and semantic information is an essential aspect of complex social interactions. A modified version of the Implicit Association Test (IAT) called Dual Valence Association Task (DVAT) was designed in order to measure conflict resolution processing from compatibility/incompatibly of semantic and facial valence. The DVAT involves two emotional valence evaluative tasks which elicits two forms of emotional compatible/incompatible associations (facial and semantic).

**Methods:**

Behavioural measures and Event Related Potentials were recorded while participants performed the DVAT.

**Results:**

Behavioural data showed a robust effect that distinguished compatible/incompatible tasks. The effects of valence and contextual association (between facial and semantic stimuli) showed early discrimination in N170 of faces. The LPP component was modulated by the compatibility of the DVAT.

**Conclusions:**

Results suggest that DVAT is a robust paradigm for studying the emotional interference effect in the processing of simultaneous information from semantic and facial stimuli.

## Background

Integrating information about emotional valence from face expressions and semantic information is an essential aspect of social interactions. In particular, the integration of emotional cues in a highly associative context (e.g., face to face communication) is critical for understanding complex social cues. For example, to understand an irony, one benefits from integrating semantic information with facial clues that orient the listener to the opposite meaning. Language modulates the information presented in facial expressions [1], and in turn, emotion modulates semantic understanding [2]. In certain situations, the incompatibility of emotional cues regarding semantic information in an associative context requires cognitive processes in order to solve this conflict. In cognitive sciences, several paradigms are considered robust indexes of the degree of conflict, such as Simon effect, or interference between routes of divergent/convergent emotional information, such as Emotional Stroop effect. Conflict tasks, also known as interference tasks, present to the subject two or more tasks to be performed simultaneously. Each task requires the implementation of a limited number of maneuvers, which produces interference or conflict when one task is incongruent with another one.

Here we present behavioural and neural correlates of an interference task, triggered by incongruent emotional discrimination, in a similar vein than the emotional Stroop task. Nevertheless, in the DVAT the interference depends on the multimodal integration of (a) configurative and emotional aspects of face processing on one hand, and (b) semantic effects on the other.

### *Implicit Association Task *(IAT)

The IAT [3] is an experimental method that measures the strength of associations between two categories, and it has been extensively used in social psychology [4,5]. The IAT is a simultaneous stimulus categorization task that works by comparing subjects' reaction times when classifying a word or image shown on the computer screen into one of two categories of response. The IAT is implicit in the sense that subjects are not directly asked about the associations, but their reaction times to these associations are measured. The IAT has been proven useful in assessing social bias by considering valence categories (i.e., varying from positive to negative) as well as social categories (e.g., Black and White people) and measuring whether experimental blocks that require "compatible" associations (i.e., in prejudice: Black people/negative and White people/positive) show higher or lower reaction times relative to "incompatible" associations (i.e., Black people/positive and White people/negative).

### Dual Valence Association Task (DVAT): An IAT measure of emotional interference

In this investigation, we designed a modified version of the IAT called the Dual Valence Association Task (DVAT). The DVAT measures interference effects presented in a double associative categorization valence tasks (that is, congruency/incongruence between emotional information presented in faces expressions and semantic information presented in words). In the DVAT, we do not use an evaluative task (e.g., positive vs. negative) and a categorization task (e.g., Black people vs. White people), but instead, we use two emotional valence evaluative tasks. Participants are asked to categorize the emotional valence of positive/negative words or the emotional valence of positive/negative faces. Both attributes, the semantic dimension (pleasant or unpleasant words) and the facial dimension (happy or angry facial expressions), must be categorized in blocks for one dimension of compatible/incompatible valence. In this way, the incompatible blocks imply either an associative contextual effect of semantic interference with facial evaluation or an associative contextual effect of facial interference with semantic evaluation.

The goal of this paper consists of providing a behavioral and ERP task for facial and semantic valence effects. The interaction between semantic and facial cues is an emerging research agenda (see review: Barret et al.[1]). Our paradigm provides a method borrowed from attitudes research (implicit association task) applied to basic facial-semantic valence interference effects.

### Early and Late ERP processing

The technique of ERPs is a precise tool regarding time resolution (in the order of milliseconds) that incorporates the recording of ongoing electrophysiological activity using electroencephalography (EEG). ERPs result from the synchronous activation of neural subpopulations that occur in response to events (sensory, motor or cognitive). ERPs are the sum of the activity of excitatory postsynaptic potential and inhibitory postsynaptic potential activated in response to each new stimulus. ERPs have a very low spatial resolution compared to neuroimaging techniques. Nevertheless, they are useful not only for their excellent temporal resolution but because recent advances (e.g., dense arrays) provides multiple sources of brain activity in response to cognitive events.

The current research of ERPs in different models of attention and emotion processes has highlighted the role of early and late cortical dynamics. Early responses (eg., 100-200 m ms after stimulus onset) index bottom-up sensory mechanisms sensitive to stimulus salience. In this regard, early modulation refers to the facilitation of early automatic and pre-attentional discrimination of salient stimuli. Later stages (300-800 ms) may be considered a marker of top-down control mechanisms that support the processing of task-relevant stimuli. The late process can be understood as a correlate of arousal and awareness triggered by the emotional content of stimuli. Moreover, it has been related to the integration of emotional stimuli features into the current context via controlled processing. Thus, the early/late processes can be understood as an early-automatic and late-controlled parallel process triggered by the stimuli salience and relevance.

Recent ERP paradigms using stimuli such as words and faces have elicited both early and late emotional effects. ERP research has demonstrated that early cerebral modulation (130-170 ms) elicited by images of faces and other objects occurs in the occipito-temporal scalp area (N170; [6,7]). Other stimuli, unlike faces and words, do not typically trigger the N170 effect [8-10]. N170 is localized to the right hemisphere for faces, and to the left hemisphere for words ( [11,12], see also [13] ).

Other studies have pointed out that N170 is modulated by faces and emotions [14-17]; but the specific direction of emotion effects is not consistent between those studies, depending on the different emotions included, tasks, and conditions. Nevertheless, when positive vs. negative valence is considered, an enhancement of positive valence amplitude has been reported with emotional pictures in early ERPs [18] and with emotional faces paradigms in N170 [19].

Contextual information (i.e., emotional background congruent or incongruent with emotional expression of faces) can also modulate N170 amplitude [20-22]. In summary, N170 is a neural marker of early face-selective processing which is not influenced by other objects processing. N170 is modulated by emotional context, affective valence and contextual cues.

Another early ERP component (which is occasionally present along with N170), is characterized by a negative voltage pattern at temporo-occipital areas of the scalp (at 200-300 ms). This component is lateralized in the left hemisphere and specifically triggered by word stimuli. This negativity is sensitive to the orthographic nature of the stimuli, their lexical/phonologic properties [13,23], but also to their semantic effects [24]. Since its semantic properties, this component would be modulated by emotional words and by semantic interference effects in the DVAT.

Late positive potential (LPP) have been related to evaluative categorization and stimulus valence [25,26]. Several studies have suggested that factors related to perceptual processing of emotional stimuli are likely to influence early ERP components, but not LPP in terms of valence [27]. In line with these findings, several studies have demonstrated that LPP is, in fact, more closely associated to arousal and higher cognitive processes relative to the specific valence of an emotional sign [28].

Similarly, previous ERP studies of comparable paradigms (e.g., lexical decision tasks with emotional stimuli, IAT tasks) have indicated an early modulation based on valence and contextual cues (N170) and a late modulation responding to incongruent blocks [18,29-32]. To sum up, early and late effects that have been reported using emotional stimuli which are characterized by an early valence effect (140-300 ms) and a later LPP modulation (300-700 ms) based on arousal and higher order cognitive process, such as evaluations between categories [33-35].

In this study, our aim was to present the DVAT as a new task designed to assess emotional interference effect using facial and semantic stimuli. We measured the behavioural and electrophysiological correlates of the DVAT. We expected to find a behavioural effect in the DVAT (i.e., longer RTs for incompatible blocks relative to compatible blocks). Following previous result described above, we expected to find specific early and late ERP effects, that is, (a) modulation of N170 for faces by valence and possible contextual association effects, but not compatibility; b) modulation of the N240-280 component for word valence in the left posterior area of the scalp upon presentations of words; and (c) modulation of a late frontal LPP effect by compatibility, but not by the specific valence of the target.

## Methods

### Participants

The participants were 20 healthy volunteers, aged between 19 and 26 years [M = 23.06, SD = 0.65], comprising of 11 males and 9 females. The sample was obtained from undergraduate student volunteers from the Cognitive Neuroscience Laboratory. All subjects participated voluntarily and signed an informed consent in agreement with the Helsinki declaration. All experimental procedures were approved by the University Ethics Committee. A questionnaire was given to all participants to rule out hearing, visual, psychiatric or neurological deficits.

### Stimuli validation

Pictorial stimuli for happy and angry faces were taken from a previous dataset used in previous studies [31,36,37]. Happy and angry facial expressions, opposite in terms of valence dimension, were selected [38]. A set of 20 happy and 20 angry pictures controlled for intensity, brightness, colour and contrast was chosen. The happy and angry sets of pictures depicted the same people. We consider happy and angry facial emotion since we have previously reported consistent effects of N170 amplitude modulation indexed by those both emotions [36,37]

Opposed valence words controlled for arousal, content, length and frequencies were also selected from a dataset reported in other study [31]. To validate word content, a questionnaire was used to gauge the pleasantness or unpleasantness of a list of 150 words with a moderate frequency use as selected using the Lifcach frequency software. A sample of 50 psychology students participated in the validation. All participants were at secondary school education or higher (average time at school 17 years, SD = 3.75), and 42 were right-handedness. The average age was 19.62 (SD = 3.33), and 67.3% were female, with no visual deficits. Participants rated the set of words using a Likert scale where 1 represented a very positive valence and 7 represented a very negative valence. A repeated measure Analysis of Variance (ANOVA) was used to contrast categorizations for the list of pleasant and unpleasant words. Important differences were obtained for the categorization of both lists [F(1, 73) = 25.16, p < 0.0001]. From the list of pleasant words, only those that were ranked between 1 and 3 were chosen (72 positive words were chosen, 3 rejected). From the list of unpleasant words only those rated between 5 and 7 were chosen (71 negative words were chosen, 4 rejected).

For faces, arousal mean value (range: 0 to 10) was 6.01 (SD = 1.23) and valence (range:-5 to 5) 0.21 (SD= 3.83). For words, arousal mean values (range: 0 to 10) was 4.94(SD = 1.83) and valence (range: -5 to 5) 0.42 (SD= 4.28) respectively. For training blocks (see below) other facial and semantic stimuli was considered.

### DVAT procedure

The task consisted of classifying faces and words as either positive or negative in valence. Behavioral responses were generated by pressing one of two keys with each forefinger (1 vs. 2). Therefore, each valence category was assigned to a different side (left vs. right). The stimuli set had 20 pictures of happy faces, 20 pictures of angry faces, 71 pleasant words and 71 unpleasant words. A greater number of word stimuli relative to faces were selected to reduce the repetition effect of words [39,32], (see discussion, 'ERP results' section). The task was organized in four different test blocks. In each block, trials were presented one by one with strict alternation between words and faces. Four labels were always visible at the top corners of the screen, two on each side, to remind the participants which answer corresponded to which response category. Each corner had a face category label (either "happy" or "angry") and a word category label (either "pleasant" or "unpleasant"). Test blocks only differed with respect to which side of the screen each category was assigned. Each test block was preceded by three practice blocks: one each for training face and word categorization and one consisting of a task identical to the corresponding test block but shorter in length. Only one pair of labels was displayed at the top corners when only one type of stimulus was presented in a training block.

In block 1 (faces training), participants categorized faces either as happy or angry. In block 2 (words training), they were asked to categorize words either as pleasant or unpleasant. For block 3 (faces/words training), evaluative categories of blocks 1 and 2 were pooled together in a single task of combined classification, such that stimuli were categorized as Angry-Unpleasant (left) or Happy-Pleasant (right). The discrimination task that was carried out in practice block 3 was repeated but with more stimuli in block 4 (test block, "Compatible"). In block 5 (words training), words were categorized in the same manner, but this time the word categories were assigned to the opposite side of the screen (face expression labels were next to incompatible semantic labels). In block 6 (faces training), faces were categorized without switching the side of the category presentation. The combined classification tasks followed in blocks 7 (faces/words training) and 8 (test block, "Incompatible"), with categories labelled on the same sides as in the preceding blocks 5 and 6: Angry-Pleasant (left) and Happy-Unpleasant (right). To counterbalance the assignment of hands to categories, the series of 8 blocks was repeated, switching sides for all labels, thus producing a total of 16 blocks.

Blocks 4 and 12 (200 trials in total) were considered compatible since both negative categories were on one side (left) and both positive categories were on the other side (right). Hence, blocks 8 and 16 (also 200 total trials), in which each side of the screen combined positive and negative classifications, were considered incompatible and were expected to be more difficult tasks. This compatible versus incompatible distinction is analogous to the one made for the IAT [3-5,40,41]. Since stimuli are the same in different test blocks, categorization is subject only to contextual manipulation through category assignment to sides of the screen. Training blocks included facial and semantic stimuli different than those presented during tests blocks.

Initially, participants were informed that the study assessed emotions and classification processes regarding faces and words. They were told that they would see words and faces and their task would consist of classifying them as having either a positive or negative valence. Instructions indicated that participant should respond as quickly as possible. Participants sat in front of a computer with electrodes placed on their heads and completed the task as the stimuli were displayed on the computer screen. On-screen instructions preceded each block to indicate what kind of stimuli would be presented and which button to press for each response category. The ERPs were stimuli locked to face and word onset (see the section on EEG recordings). Once the experiment had finished, participants were thanked, and the research goal was thoroughly explained.

Practice blocks involved 20 stimuli, consisting of 10 face and 10 word stimuli, and test blocks comprised 100 stimuli, that is, 50 face stimuli and 50 word stimuli. Based on previous reports [31,32], and to ensure conscious access to faces and words, the time window during which stimuli were presented was set at 100 ms (faces) and 300 ms (words). Research on behavioral and ERP assessment of word processing usually presents stimuli for 300 ms or more (e.g., [42-45]). When words are presented for less than 300 ms, early effects are difficult to obtain and an increase in error rates is expected. A recent report found an early (N170) effect when stimuli duration was as long as response time [46]. On the contrary, facial stimuli requires less time for conscious detection and processing (e.g., around 100 ms: [16,47,48].

Stimuli (faces and words) were repeated in order to obtain an enough number of trials for ERP estimation. Stimuli (faces and words) were repeated in order to obtain a sufficient number of trials for ERP estimation [32,39]. The repetition effect of words is a robust modulator of ERPs [49-58]. On the contrary, facial ERP modulation can be found even in a reduced number of faces (e.g., [53-58]. Moreover, the habituation effects (e.g., attenuation of N170 amplitude and non-lateralization effects) are mostly frequent when the stimuli consist only of faces. When facial stimuli is presented together with other non facial stimulus (e.g., objects, words) the habituation and repetition effects disappear [58]. Given the mixed and counterbalanced presentation of faces and words in our paradigm, we chose a small number of faces (40) because we do not expect habituation effects.

**Figure 1:**
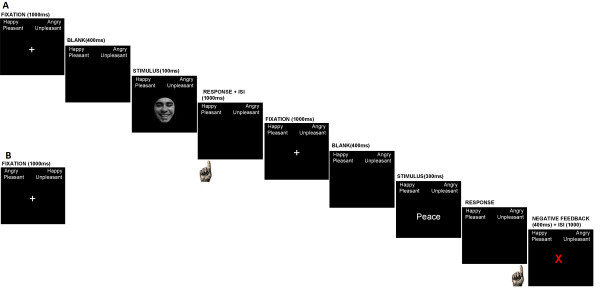
**Sequence representation for compatible test block**. A. The schema shows a face and a word sequence presentation and the participant's response. Face and word trials are alternately presented for a short time, strictly. Positive and negative faces, along with words of positive and negative valence, are present in the stimuli set and are presented in a randomized sequence. The participant is required to classify each stimulus to the left or to the right according to labels displayed on top of the screen. When a classification response error is made, negative feedback is given (i.e., in the word sequence illustrated in the figure). B. During the incompatible block test the valence classification of a face (e.g., happy-left vs. angry-right) must be classified in the opposite valence direction of the word (unpleasant-left vs. pleasant-right). When items from compatible categories (e.g. happy + pleasant) share a response key, performance is faster and more accurate than when items from incongruent categories (e.g. angry + pleasant) share a key

Figure 1 illustrates an example of a compatible test block. Each trial began with a fixation trial (1000 ms), followed by a blank screen (400 ms) and then a word (300 ms) or a face (100 ms) in strict alternation. Following face or word presentation, participants had to categorize the valence of the stimulus displayed on the computer screen into one of two response categories (positive or negative). Incorrect responses were indicated with an 'X' (400 ms) in the center of the screen immediately after the response (however participants did not have to correct the error made after the 'X' appeared). The negative feedback was introduced to ensure the subject paid attention throughout the task. Then, an ISI of 1000 ms was introduced. The stimuli were presented in the center of a black background of a 17-in color monitor. Each stimulus was centered horizontally and vertically on the screen subtending a visual angle of 4.5° × 3.15° at a viewing distance of approximately 80 cm.

### EEG Recordings

EEG signals were sampled at 500 Hz from 129-channel system with HydroCel Sensors from an Electrical Geodesic amplifier. Data outside the frequency band that ranged from 0.1 Hz to 100 Hz were filtered out during the recording. Later, a band pass digital filter between 0.5 and 30 Hz was applied to remove unwanted frequency components. During recording the reference was set by default to vertex but then was re-referenced off-line to average electrodes. Two bipolar derivations were designed to monitor vertical and horizontal ocular movements (EOG). ERPs were trigged from stimulus onset (faces and words).Continuous EEG data were segmented from 200 ms prior to the stimulus to 800 ms after the stimulus. All segments with eye movement contamination were removed from further analysis using an automatic (Gratton, Coles, and Donchin method for removing eye-blink artifacts) and visual procedure. Artifact-free segments were averaged to obtain ERPs.

### Data analysis

#### DVAT score computing

A DVAT score was calculated for each subject based on reaction times obtained from the test. This numeric value provides an index of difference between compatible and incompatible tasks. Greenwald, Nosek, & Banaji [59] proposed a method to calculate this rate for social categorization IATs, which involves eliminating extreme reaction times, special management of wrong responses and standardization of resulting reaction times based on the standard of each subject. In brief, this method eliminates reaction times over 10000 ms; however, since our task was a rapid forced-choice task, we eliminated responses with more than the 2000 ms from the analysis. Finally the method recalculates wrong answers (error trials) by adding 600 ms to their real value. Miss trials (trials in which the subject does not respond), are not included in the analysis. A subject's score is obtained by measuring the reaction time difference between compatible and incompatible blocks, standardized according to the standard deviation (See additional file 1 for a detailed explanation of the method).This method constitutes the best procedure for two-choice compatible/incompatible tasks since it reduces the method-specific variance [60]. Hence, the measurement procedure was evaluated for bias using a scale that enables a comparison between different subjects. The result of this procedure was a number with an expected value close to zero for subjects who did not show any bias in the test. Negative values corresponded to the detection of bias in favour of the compatible task. In addition, as reported in the supplementary data (additional file 2), we did the same analysis excluding the error trials and the penalties. Similar effects were obtained with this procedure. Finally, in the supplementary data (additional file 2) the accuracy as well as the reaction times of each category was included.

#### ERP analysis

Matlab software, EEGLab toolbox and T-BESP software (http://www.neuro.udp.cl/) were used for off-line processing and analysis of EEG data. Regions of interest (ROIs) were used to analyze the scalp topography of the ERP components [61], which is recommended for dense arrays since it improves statistical power. ROIs were chosen by visual inspection of each component. Each N170 ROI (and for the left occipito-temporal negativity) consisted of three adjacent electrodes (see additional file 3 for those channel locations): the N170 ROIs were 65, 69, and 70 for the left and 83, 89, and 90 for the right. Frontal LPP was estimated based on previously published electrode localizations [25]: Left (22, 23, 25, 26) and Right (2, 3, 8, 9). Although signal plots show the overall averages of ERPs for each data cell, statistical tests were performed separately considering data for each participant using R software (http://www.r-project.org). For ERP analysis, mean average amplitudes were considered. The 140-190 ms time window for N170; 240-280 ms time window for the left occipito-temporal negativity; and the 400-700 ms time window for LPP were visually selected for mean amplitude analysis.

The main factors submitted for analysis were *task, valence, *and *contextual association*. ERP waveforms were averaged for faces and words separately and analyzed using a repeated measure ANOVA with Task (compatible and incompatible) as the within-subject factor. This factor involves two levels of word-face association: a compatible task (happiness/pleasant association and anger/unpleasant association) and an incompatible task (happiness/unpleasant association and anger/pleasant association).

For each stimulus type (face and word), Task was defined as follow: Compatible tasks for faces (faces associated with words of the same valence--[face positive/word positive] and [face negative/word negative]) were compared to incompatible tasks (face associated with words of the opposite valence--[face negative/word positive] and [face positive/word negative]). Following the same procedure, compatible task for words (words associated with faces of the same valence--[word positive/face positive] and [word negative/face negative]) were compared to incompatible tasks (words associated with faces of the opposite valence--[word negative/face positive] and [word positive/face negative]).

To compare the specific effects of the target valence as well as the associated valence of the contextual affects, ERP waveforms for early and late ERP effects were averaged for faces and words separately and analyzed using a repeated measure ANOVA with the following within-subject factors: valence (positive vs. negative) and contextual association, which involves two possible context associations (positive and negative) for both faces and words. Faces (targets) were categorized in positive or negative valences of word contextual association (context of the task). At the same time, words (targets) were categorized in positive or negative valences of facial contextual association (context of the task). Interactions between valence and contextual association produce four categories (for both faces and words targets): positive target in a positive context; positive target in a negative context; negative target in a positive context; and negative target in a negative context. Finally, for each component, the factor Hemisphere (N170 left and right locations) was considered.

Partial eta squared (η^2^) effect size measures were computed for significant effects and relevant trends. Univariate comparisons were done whenever necessary. Results were corrected with the Greenhouse-Geisser and Bonferroni's methods to adjust the univariate output of repeated measure ANOVAs for violations of the compound symmetry assumption.

## Results

### Behavioural data: performance and DVAT scores

#### Accuracy and reaction times

No participants were eliminated based on Greenwald et al.' 2003 criterion of 75% accuracy on each block [59]. This percentage was obtained considering only correct responses within the 2000 ms (excluding errors and absent responses). Participants performed over >80% of accuracy in all categories. Regarding reaction times, no effects of valence or contextual association were found (see table 1 for accuracy and RTs descriptive statistic). Nevertheless, facial and semantic stimuli elicited a task effect: Compatible blocks produced shorter RTs than incompatible ones. Accuracy and RTS effects are presented in the supplementary data (additional file 2).

**Table 1:** Descriptive statistics of DVAT (accuracy and reaction times).

Category	Accuracy (%)		Reaction Times (ms)	
	
	*M*	*SD*	*M*	*SD*
*face- positive + context positive*	93	3,6	890	221
*face- negative + context positive*	85	6,7	970	243
*face- positive + context negative*	91	5,6	1187	283
*face- negative + context negative*	87	3,8	1140	212
*word- positive + context positive*	94	4,2	1220	287
*word- negative + context positive*	83	5,3	1662	287
*word- positive + context negative*	81	5,6	1634	298
*word- negative + context negative*	92	4,4	1303	245

Total	88,25	4,9	1250	258

#### DVAT scores

Mean DVAT score of the subjects was -1.67 [SD = 1.03], a value significantly less than zero [t(20) = -7.39, p = 0.0003, Cohen's d=-1.61]. DVAT score provides an index of difference between compatible and incompatible tasks (a value close to zero for subjects indicated no DVAT effects of compatibility). Only one subject obtained a non-negative DVAT score (value= 0.07). Thus, the reaction times of the subjects were longer on average in the incompatible blocks relative to the compatible blocks. Our results show, as expected, that the compatible valence blocks (happy/pleasant and anger/unpleasant association) were facilitated, compared to the incompatible valence blocks (happy/unpleasant and anger/pleasant association).

In our sample, DVAT scores had a Spearman-Brown corrected split-half reliability of r_SB _= 0.82, yielding a standard error of σ_err _= 0.44. Split-half reliability was computed considering each picture trial and the following word trial as an atomic unit. All units were used in an odd-even reliability estimation. In other words, each half had words and pictures, the same structure, and was the same for every subject. This result suggests that the DVAT yields high reliability measurements, and provides an estimation of the standard error of the test making it useful for distinguishing actual differences from measurement error. We performed an additional DVAT score analysis, this time excluding errors and penalties (see supplementary data in additional file 2). Those results were similar to the DVAT scores calculation presented here.

### N170 and early effects

**Figure 2:**
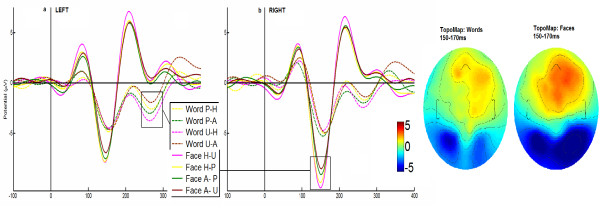
**N170 waveform and topography**. N170 component elicited by emotional words and faces. The N170 modulation is predominant in the left hemisphere for words and in the right for faces. Boxes in ERPs figures are indicative of regions where statistical comparison shown significant differences. Right side of the figure: Word and Face voltage maps averaged on 140-190 ms. Abbreviations: Word P-A (pleasant words associated to anger face); Word U-A (unpleasant words associated to anger face); Face H-P (happy faces associated to pleasant word); Face H-U (happy faces associated to unpleasant word); Face A-U (anger faces associated to unpleasant word); Face A-P (anger faces associated to pleasant word).

For the N170 elicited by faces, a main effect of Valence was observed [F(1, 19) = 12.23; p = 0.005; partial η^2 ^= 0.39]. The N170 showed a larger amplitude in the positive valence condition [M = -8.63, SD = 1.84] compared to the negative valence condition [M = -7.51,SD = 1.72]. In line with this observation, an interaction effect between Valence and Hemisphere was observed [F(1, 19) = 5.80; p = 0.03; partial η^2 ^= 0.23]. Post-hoc comparisons [HSD test, MS = 2.42; df = 12,00] showed that positive valence faces [M = -9.86, SD = 1.85] elicited more amplitude than negative valence faces [M = -8,007, SD = 1.72] in the right hemisphere [p < 0.005]. In addition, an interaction between the Valence and Contextual Association was observed [F(1, 19) = 7.43, p < 0.05; partial η^2 ^= 0.28]. Post-hoc comparisons carried out for this interaction (see figure 2.b) suggested that only the category with the largest modulation, that is happy faces associated with unpleasant words [M = -8.93, SD = 1.94], was distinguished from angry faces associated with unpleasant words [M = -7.06, SD = 1.66, p = 0.02;HSD test, MS = 4.15, df = 12.00]. No effects of *Task *compatibility were found.

The N170 elicited by words was not significantly affected by any of the relevant factors (see figure 2.a). However, in the left region of the scalp a second negative peak was observed (aprox. 270 ms). This component appeared to behave partially like the N170 effect in the right hemisphere observed with facial stimuli. Nonetheless, no main effects of Valence were found and only a trend between Valence and Contextual association interaction was observed [F(1, 19) = 4.12, p = 0.06; partial η^2 ^= 0.18]. Post-hoc comparisons [HSD test, MS = 3.95, df = 12.00] carried out for this interaction suggested that only the category with the largest modulation, unpleasant words associated with happy faces, [M = -2.98, SD = 0.56], was distinguished from unpleasant words associated with angry faces [M = -1.23, SD = 0.47, p = 0.02]. As with the N170 component, no effects of Task compatibility were found in this time window.

### LPP late effects

Regarding faces and words, no effects were found for *Valence, Contextual association *or *Hemisphere*. Nevertheless, a *Task *compatibility effect was present for both faces and words.

**Figure 3:**
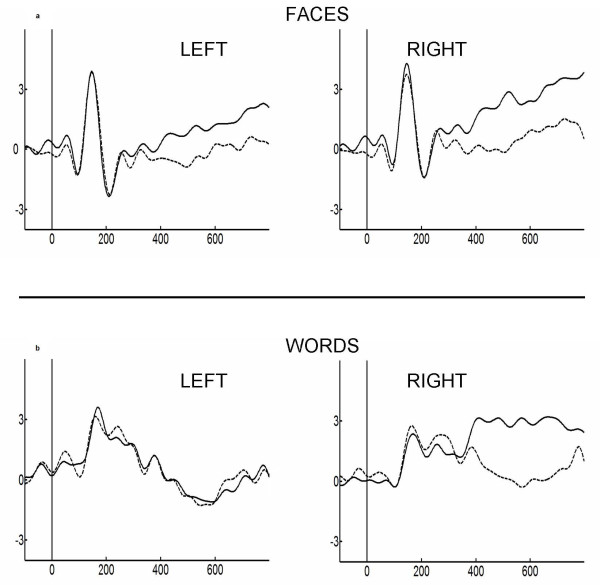
**Frontal LPP**. Selected ROIs of LPP elicited by faces and words in response to Incompatible (continuous line) and Compatible tasks (dashed line). Top: ERPs of Faces. Bottom: ERPs of Words. In agreement with the behavioral results, we found a LPP modulation related to incompatible blocks. Note the lateralization effects for word stimuli and the bilateral effects for faces.

In the *Task *comparison for faces, a main effect of Hemisphere was found [F(1, 19) = 20.73, p = 0.0006; partial η^2 ^= 0.52], and only a trend for the effect of Task was found [F(1, 19) = 3.51, p = 0.085; partial η^2 ^= 0.16]. Nevertheless, an interaction between Task and Hemisphere was observed [F(1,19) = 9.31, p < 0.01; partial η^2 ^= 0.33]. Post-hoc comparisons for this interaction [HSD test, MS = 0.44 df = 12.000] found larger amplitudes and differences between compatible [M = 1.15, SD = 0.44] and incompatible [M = 3.48, SD = 0.82] blocks in the right hemisphere [p = 0.0002]. In the left hemisphere, a small but still significant difference was also observed between compatible [M = -0.34, SD = 0.62] and incompatible [M = 0.98, SD = 0.76] blocks [p < 0.05]. In both hemispheres, the incompatible blocks elicited larger amplitudes relative to compatible blocks (see figure 3.a).

For words, a main effect of Hemisphere was found [F(1, 19)= 9.99, p = 0.008; partial η^2 ^= 0.35], but no main effect of Task was found. Nevertheless, an interaction between Task and Hemisphere, similar to our faces effect but slightly stronger, was observed [F(1, 19) = 8.81, p = 0.01; partial η^2 ^= 0.32]. Post-hoc comparisons for this interaction [HSD test, MS = 1.34, df = 12.00] found only differences between the compatible [M = 0.49, SD = 0.82] and incompatible [M = 2.82, SD = 0.65] blocks in the right hemisphere [p = 0.0002]. No differences were found in the left hemisphere (see figure 3.b).

Synthesizing the ERP main results, the effects of valence and contextual association were observed at an early processing (N170 component) but not later. Compatibility effects were observed only at later process as indexed by the LPP component.

## Discussion

The aim of this study was to examine performance in a DVAT with two forms of emotional compatible/incompatible associations (facial and semantic). This novel task, although similar to IAT, does not involve an evaluative dimension and a categorization task but a bimodal (faces and words) emotional conflict task. This paradigm evaluates the emotional conflict and facilitation of incompatible and compatible bimodal valence associations, suggesting that this modified IAT is suitable for emotional interference research on normal and psychiatric populations.

### The Novel Relevance of DVAT: Implicit cognition and Conflict tasks

Several recent studies investigating IAT have criticized behavioural measurements based on classical bias attributes in incompatibility effects. There is an ongoing debate about its significance as an attitude index, largely due to theoretical assumptions about the cognitive and behavioural processes involved in an IAT task [40,41,62-65]. IAT may not measure only social bias, but also several other cognitive processes [4], for example, interference effects based on stimuli. Although our results do not provide data to directly address this issue, they support this hypothesis. Since DVAT uses two evaluative judgments, the incompatibility effects might be explained by an interference effect that forces a complex strategy: for incompatible blocks, a dual strategy (i.e., positive word valence in one direction and positive face valence in another direction) is expected to demand more effort and time in comparison to a simple strategy (ie., words and pictures valence in the same direction). This effect is relevant to the hypothesis that the IAT is a dual task (a simple and a complex task with interference), given that no social bias effect or similar effect is present in our task. Furthermore, our behavioural results show that the pattern of the IAT effect is present in social bias paradigms. In race-IAT, this process affects IAT scores. For example, when participants showing implicit racial bias performed a race-IAT, in the compatible blocks (White-positive task), they simply responded to both faces and words on the basis of whether they liked the presented stimuli. Because task switching leads to performance costs, performance is diminished in the Black-positive task compared to the White-positive task (See [4] for a detailed discussion on this topic). Thus, task switching in the more complex task (incompatible) would be mainly triggered by this interference.

In both IAT and DVAT, the "implicit" property relies in the fact that the association between faces and word content is not explicit. Nevertheless, DVAT's implicitness property has to be carefully considered. We have adopted the De Houwer definition of implicit measure as "outcome of a measurement procedure that results from automatic processes by which the to-be measured attribute causally determines the outcome" [66]. The DVAT can only partially assess the normative criterion of this definition (the "What" criterion, the "How" criterion and the implicitness criterion; see De Houwer et al. 66] for more details on this definition).

Our results suggest that the DVAT can be used as a conflict or interference measure, with early and late neural correlates. A classic conflict or interference paradigm that has been thoroughly studied is the Stroop effect, which resembles the classic IAT regarding compatible/incompatible tasks [41,67]. The first Stroop studies were comprised of incongruent trials in which a word referring to a colour was presented (e. g., red), but the actual color in which the word was written was incongruent (e. g., blue), provoking longer reaction times. This reaction time effect accounts for the interference provoked by the conflict emerging from the incongruence of the semantic meaning of the word and the colour in which it is written [68]. The Stroop task has undergone several modifications since it was created to examine and explain the interference phenomenon and relevant variables [69,70]. Consistent with our results, this electrophysiological interference effect appears to be modulated at later times. Recent studies suggest that the interference process occurs closer to the response stage than to the stimulus-processing stage [71]. The Simon effect [72] is another classic interference paradigm (although with no emotional content) that is observed in information processing during response selection. In an electrophysiological study of Simon effect [73], although an early modulation (N1, N2) was observed, the reaction times associated with incongruence have been found to correlate with the P300 component. In summary, the processing of interference stimulated by a pair of incongruent stimuli is a late process [68,74] that occurs during the response selection stage, which explains the longer times that test subjects need to respond to incongruent stimuli. The DVAT results are in agreement with previous reports of conflict tasks, an additionally elicits electrophysiological early (valence modulation) and a late (conflict modulation) stages of emotional integration and interference.

This is the first report of an implicit association task based on interference effects (triggered by facial and semantic valences) being more suitable for basic emotion research than IAT. Compared to the emotional Stroop effect, the interference is not triggered by colour and emotion, but by facial processing and semantic valence. Emotional inference of facial clues is one of the most important steps in the development of complex social behavior [75]. Faces are multi-dimensional stimuli directly related to important social incentives [76]. Emotional face expression gives an automatic and fast shortcut to alarm signals, metallization and inter-subjective communication. Thus, the DVAT opens a new area of research on emotional interference effects better suited for social cognition research than the emotional stroop task. DVAT considers stimuli (emotional faces) with high relevance for social cognition. Our report has shown cortical markers of early valence modulation and late compatibility effect suggesting that emotional markers and evaluative comparison are processed in two temporal brain dynamics. Valence and emotional interference would be automatically processed in the structural stage (eg., fusiform gyrus and superior temporal sulcus indexed by N170). Compatibility should be processed in a more controlled and delayed process, indexed by prefrontal cortices, as LPP topography suggests. Early emotional decoding possibly subserves adaptive social behavior; and later LPP processing indexes cognitive control and behavioral performance. Those results are consistent with recent reports of context-dependant effects on cognitive processing [29-32,77-86].

Considering that interference and conflict studies have had consistent applicability in psychiatry, the DVAT might be a sensitive paradigm for pathologies such as schizophrenia and ADHD, in which emotional and inhibition/interference deficits are simultaneously observed. Since this paradigm demonstrates the early effects of valence and the late effects of interference in electrophysiological measures, it may be used to investigate the time course of both processes in these psychiatric populations. Moreover, early and late ERPs to emotional processing are modulated by psychiatric medication [87]. Future studies would assess the possible role of psychiatric medication (antidepressants, mood stabilizers, stimulants, etc) on emotional interference effects indexed by the DVAT.

A well-known effect of blocked presentation in interference tasks (e.g., Stroop effect) has been reported elsewhere (for a recent report, see [42]. In our design, the main reason for selecting a blocked presentation (here as well as in multiple IAT tasks) is the complexity of the task: if participants have to constantly change the dual categorization of faces and words, as well as their corresponding positive and negative valence, the paradigm becomes difficult to perform. Using a blocked presentation allows the learning of the task during the practice trials. Future studies, however, with a well-designed paradigm, should test the compatibility effects in mixed blocks.

### Behavioural pattern

The accuracy was relatively high in all categories of DVAT (mean: 88%) and the reaction times were modulated only by task compatibility. Our DVAT scores showed a robust effect that distinguished compatible/incompatible tasks. Incompatible task blocks generated a longer latency in responses, supporting an interference effect (of words on pictures and vice versa) produced by two opposed pairs in the same dimension of emotional valence. Indeed, behavioural data showed a very strong DVAT effect. The mean DVAT score was negative with large statistical significance, which was expected because subjects were faster when their task consisted of an association in the same valence dimension (positive: happy/pleasant vs. negative: angry/unpleasant). This evidence suggests that the DVAT is sensitive to interference in valence associations of facial and semantic stimuli. The emotional content of the target (either a word or picture) is contextually affected by the compatibility or incompatibility of the subsidiary categorization (either word or picture) and is independent of its specific content (positive or negative). Therefore, regardless of the specific valence of the target, the responses (DVAT scores) are modulated by the interference/association or facilitation of the subsidiary category valence.

### The relation of our ERP results and previous research

Confirming our hypothesis, ERP results indicated the occurrence of early and late modulations. When presented with face stimuli, the N170 component was mainly modulated by valence (positive > negative) and showed independence from compatibility effects. Only a trend of contextual association was observed in the right scalp, which occurred for positive valence faces (happiness) associated with a negative semantic valence (unpleasant). This finding suggests an early effect on the salient categories of contextual incongruence. Nevertheless, there was no effect of this component on incompatibility.

Word ERPs were not modulated as a function of any of the variables previously described for the N170 window. However, a specific trend of word stimuli in the left hemisphere was later observed. In the 240-280 ms time window, a second negative peak was observed, which was modulated as a function of valence and contextual association (unpleasant words associated with happy stimuli > unpleasant words associated with anger stimuli). Once more, there was no compatibility effect.

Of interest, we only observed significant discrimination in the frontal LPP component window based on compatible and incompatible categories, independent of the specific valence, both for words (a restricted effect in the right scalp) and faces (both hemispheres). The LPP was affected neither by main effects of valence for face and word targets, nor by contextual association. Importantly, when compared to reaction times, LPP effects presented a similar pattern (modulation by task) confirming the association of later ERP components to behavioral reposes and arousal [33-35]

In summary, the effects of valence and contextual association showed early discrimination in ERPs of pictures and a tendency for words (N170 and the second peak around 240-280 ms, respectively). LPP component corresponded with behavioural measures, accounting for late modulation by the compatibility of the DVAT.

This study is in concordance with several previous findings. First, N170 was larger for faces than for words [8-10,88], and this effect was lateralized to the right hemisphere when pictures were used as stimuli, which has been previously observed especially when the experimental paradigm includes faces and words or faces and objects [11,12,89]. Only contextual effects of salient stimulus associations were observed [20-22], but not of incompatibility [29]. Finally, early components has been previously reported as modulated by emotional effects, showing increased amplitude from positive stimuli over neutral stimuli (EPN: [18]; N170 for faces:[19]).

In other hand, an early posterior negativity (EPN) has been described in paradigms of affective picture processing as an index of emotional discrimination [18,90]. An EPN-like has been recently reported to be elicited by faces and words [19]. We did not find such effect; however, different studies with emotional faces (for a review, [91] or words: for a review see, [92]) have not reported an EPN either. Probably, task dependent factors can explain the presence or absence of EPN.

Words showed no N170 modulation of valence content, in agreement with previously reported results [19]. However, we find a delayed (N230-280 ms) emotional modulation tendency. Its modulation in our study partially resembles the N170 effects elicited by faces, but a delayed response, maybe caused by the delayed word presentation. This early semantic modulation, probably facilitated by emotional content, has been recently reported in several paradigms [93-95]. Further studies with a manipulation of time window stimuli presentation should asses if this early modulation is part of a N170-like component.

In later stages, consistent with reaction times, frontal LPPs were exclusively modulated by the effect of incompatible blocks (more amplitude) in comparison to compatible ones. This effect was more pronounced in the right hemisphere when word stimuli where used. These results are consistent with previous studies showing a modulation of the LPP in a valence judgment task based on compatibility[31] and arousal stimuli [25]. The frontal effects are compatible with the role attributed to anterior structures in interference. The anterior cingulate cortex (ACC) has been associated with the detection of interference and its functional relationship with the prefrontal cortex [74]. Studies using fMRI have indicated that in Stroop tasks, interference activates frontal areas, such as the ACC and dorsolateral prefrontal cortex [69].

Following classical assessment of the two-choice task [96-99], negative feedback (an "X" appeared on the screen) was given immediately after incorrect categorization of a stimulus. The negative feedback was introduced to increase the attention of the participants during the task. The ISI (1000 ms) and the fixation (1000 ms) following the negative feedback prevented any overlap effect of the feedback and the following face and word stimuli. At the same time, the response (and consequently the negative feedback) overlapped after the ERP window locked to the stimulus (M = 1250 ms, SD = 258). Thus, feedback does not affect the previous face or word ERP window (0 to 700 ms, from onset to LPP). The time course of the stimuli (the ISI and the fixation by one side, and the delay in the response by other) avoids any feedback overlap with the preceding and following stimulus.

### Early and Late effects of DVAT

Various studies with emotional stimuli have indicated that modulation of early and late components (i.e., the *direction *of amplitude differences) is not consistent amongst different types of paradigms and stimulus factors. The task appears to affect differentially early and late components. Indeed, several previous ERPs studies of emotional stimuli showed different effects and alterations attributed to aspects of the stimuli and experimental setting, including picture size, presentation time, gender, inter-picture interval, inclusion of distracters, stimulus relevance, spatial frequency, colour, complexity and feature composition of pictures [27,35,100-103]. The fact that different stimuli and tasks produce different results suggests that ERP results are paradigm- dependent [104]. More importantly, it is still uncertain how various attributes of stimuli interact and how they affect the overall affective process [34,101]. Future studies should assess whether the direction of the early effects of valence and LPP components are paradigm-dependent aspects or specific properties associated with the categorization of the dual-valence task.

Despite paradigm-dependent differences in previous reports and the novelty of our results, the overall pattern confirms emotional effects previously reported with other paradigms: an early modulation based on valence and a late effect of arousal and higher order cognitive processes [33-35]; these findings contribute to the clarification of these discrepancies. The configuration aspects of stimuli seem to be discriminated early, but their effects are combined during later phases. These results are particularly similar to reported effects, where early modulation has been observed for configuration categories (i.e., positive valence) and associated contextual effects together with subsequent late modulation based on compatibility. However, unlike those reports, in the DVAT the early effects are related to the discrimination of valence, and incompatibility is recognized later as evidenced by non-lateralized frontal LPPs upon presentation of faces. Future studies must examine whether early effects (valence and context) and late effects (task compatibility/incompatibility) are caused by intrinsic emotional biases of stimuli or only dependent on the interference effect presented in a dual categorization task.

### Limitations and future testing

Extensive behavioural assessment is required in order to confirm the validity of DVAT. For future neuropsychiatric evaluation, a multivariate analysis including neuropsychological performance in combination with DVAT is desired. A larger sample would allow the study of individual differences related to the performance of this paradigm. As a new emotional interference measure, a comprehensive testing of their psychometric properties is needed (reliability, validity), as well as ERP source localization studies, and the examination of the paradigm on affective disorders.

## Conclusions

Our results suggest that the DVAT is a robust behavioural and electrophysiological paradigm for studying the emotional interference effect in the processing of simultaneous information from semantic and facial stimuli. The effects of valence (more in faces than in words) are discriminated early, and the effects of compatibility, regardless of the specific valence, are observed at a later time (consistent with reaction time effects). Future studies are required to determine the applicability of this paradigm as a conflict marker of socio-emotional skills in normal and psychiatric populations.

## Competing interests

The authors declare that they have no competing interests.

## Authors' contributions

The study was conceived and designed by AI. Data were captured and analyzed by AI, RE AL and EH. EH, AP, RR, AL, SB and AI participated in discussions about analysis and interpretation of data and wrote parts of the article. FM and EH revised the manuscript, critically contributing to its generation. All authors read and approved the final manuscript.

## Supplementary Material

Additional file 1 **Supplementary data on DVAT algorithm**. Click here for file

Additional file 2 **Supplementary data on behavioral measures**. Click here for file

Additional file 3 **Supplementary data on Channel Locations**. Click here for file
