# Supplementary material for: Facial and semantic emotional interference: A pilot study on the behavioral and cortical responses to the dual valence association task
Source: Behav Brain Funct. 2011 Apr 13;7:8. doi: 10.1186/1744-9081-7-8 (PMC3087672; doi:10.1186/1744-9081-7-8)
Supplement: Additional file 1 — Supplementary data on DVAT algorithm. [file 1744-9081-7-8-S1.DOC]

**Facial and semantic emotional interference: A pilot study on the behavioral and cortical responses to the dual valence association task**

**Additional File 1: Supplementary data on DVAT algorithm**

The following steps describe the application of the algorithm proposed in [59] to the DVAT test results relevant to this study.

1. All the data for the compatible (3 & 4) and incompatible blocks (7 & 8) was obtained from both key assignments. The same procedure was applied at the second sequence of block presentation in this and in the following steps.

2. Trails with a response time over 1000 ms were eliminated.

3. For each blocks, the reaction time average for trials with a correct response was obtained.

4. A pooled standard deviation was calculated for all block 3 and 7 trials, and then another for blocks 4 and 8.

5. For each trial with an incorrect response, the reaction time was replaced by the corresponding block average (counted in step 3) plus 600 ms. This corresponds to a penalty applied to wrong responses.

6. Based on the resulting reaction times, both correct and incorrect trials, the average reaction time was calculated for each of the four blocks.

7. The average difference was calculated between block 7 and 3, and another difference between block 8 and 4.

8. Each average was divided by the corresponding pooled standard deviation (counted in step 4).

9. The DVAT score is the average of the two values obtained in the previous step.
