# Supplementary material for: Facial and semantic emotional interference: A pilot study on the behavioral and cortical responses to the dual valence association task
Source: Behav Brain Funct. 2011 Apr 13;7:8. doi: 10.1186/1744-9081-7-8 (PMC3087672; doi:10.1186/1744-9081-7-8)
Supplement: Additional file 2 — Supplementary data on behavioral measures. [file 1744-9081-7-8-S2.DOC]

**Facial and semantic emotional interference: A pilot study on the behavioral and cortical responses to the dual valence association task**

**Additional File 2: Supplementary data on behavioral measures**

Accuracy.

No participants were eliminated based on Greenwald et al’ criterion of 75% accuracy on each subcategory [59]. Participants performed over 80% of accuracy. Table 1 present the means and SD for each category.

Reaction Times

Reaction times for all categories are presented in table 1. For face stimuli, no effects of valence or contextual association were found. Nevertheless, a task effect was found (F(1, 18)=9.43, p<0.05), evidencing that compatible category elicited shorter RTs. Similarly, for words, no valence or contextual association effects were found, but an effect of task yield significant differences (F(1, 18)=11.72, p<0.01). Similar to faces, compatible block of words produced shorter RTs than incompatible one. Note that the task effects results are consistent with the DVAT scores, as well as with the LPP effects for words and faces.

DVAT scores (excluding errors and penalties)

When we re-analyzed the DVAT score excluding errors and penalties from the algorithm, again a value significantly different of zero was found [t(20)=-6.89, p<0.001]. The mean DVTA score was -1.34 [SD=1.19]. Our result shows similar effects than DVAT scores performed with the canonical procedure [59]: longer RTs in the incompatible blocks relative to the compatible blocks.
