# Supplementary material for: Facial and semantic emotional interference: A pilot study on the behavioral and cortical responses to the dual valence association task
Source: Behav Brain Funct. 2011 Apr 13;7:8. doi: 10.1186/1744-9081-7-8 (PMC3087672; doi:10.1186/1744-9081-7-8)
Supplement: Additional file 3 — Supplementary data on Channel Locations. [file 1744-9081-7-8-S3.DOCX]

**Facial and semantic emotional interference: A pilot study on the behavioral and cortical responses to the dual valence association task**

**Additional File 3: Supplementary data on Channel Locations**

**Channel locations for electrode net**. Figure shows a schematic representation of electrode localizations (EGI HCGSN128 net) over the scalp.
